# Effect-Based Assessment of the Quality and Potential Presence of Hazardous Chemical Pollutants in Drinking and Potable Water in Mexico City

**DOI:** 10.1021/acsestwater.5c01058

**Published:** 2025-12-29

**Authors:** Aline Colonnello Montero, Geeta Mandava, Johan Lundqvist

**Affiliations:** Department of Animal Biosciences, Faculty of Veterinary Medicine and Animal Science, 8095 Swedish University of Agricultural Sciences, 756 51 Uppsala, Sweden

**Keywords:** effect-based methods, toxicological end points, bioactivity, bioanalytical equivalent concentrations, effect-based trigger values, water quality

## Abstract

Effect-based methods (EBMs) are bioanalytical tools detecting bioactivity of chemical mixtures on different toxicological end points. EBMs have become increasingly important for water quality assessment and monitoring, particularly in Europe and Australia. To date, their application has not been reported for the assessment of water in Mexico, where tap water is often not consumed as drinking water due to perceived concerns of pollution from the distribution system. In this study, a battery of EBMs was applied to assess the quality of drinking and potable water from Mexico City and surrounding states. The results were compared with international reports and proposed effect-based trigger (EBTs) values. Aryl hydrocarbon receptor bioactivity and androgen receptor (AR) inhibition were detected in tap water and household-filtered water. Estrogen receptor activity was observed in most of the samples, with the highest levels detected in water from the jug container. No bioactivities were detected for AR activity, genotoxicity, or oxidative stress. Although some of the samples were bioactive, the calculated bioanalytical equivalent concentrations (BEQs) were generally below the reported BEQs from other countries and below the proposed EBTs for drinking water. These findings indicate that the tested drinking and potable water sources in the surrounding states of Mexico City are of good quality.

## Graphical abstract

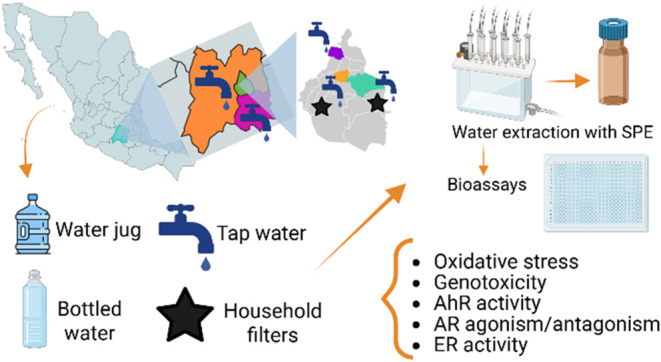

## 1 Introduction

On a global scale, freshwater sources used to produce water destined for human consumption are subject to pollution from a variety of anthropogenic substances due to increasing agricultural expansion and urban growth.^1,2^ The presence of thousands of chemicals in water bodies may pose a risk to human health given that some of these can be hazardous and exert their toxicity at very low concentrations.^3^ Moreover, the presence of unknown chemicals and the mixture effects of multiple chemicals with similar modes of action represent an additional threat to water safety.^4^

To date, the water quality assessment of source and finished waters relies mainly on targeted chemical analysis. However, this detection method detects only a small portion of the totality of chemicals present in water. Moreover, targeted chemical analysis does not account for mixture effects or the contribution of bioactive chemicals occurring below the chemical limit of detection.^5^ For these reasons, the application of effect-based methods (EBMs) as a complementary tool to chemical analysis for water quality assessment is imperative. EBMs are *in vitro* bioassays based on cultured cells assessing the mixture effects of chemicals present in water that exert similar modes of action.^6^ These effects are assessed for different toxicological end points such as activation or inhibition of hormone receptors (androgen and estrogen), oxidative stress (Nrf2 antioxidant defense pathway), xenobiotic metabolism (aryl hydrocarbon receptor [AhR] activation), and genotoxicity.^7,8^ Multiple reports^7,9–12^ have demonstrated that only a small fraction of the biological effects observed in bioassays involving xenobiotic metabolism can be explained by the chemicals detected with targeted chemical analysis, whereas the remaining effects are attributed to unknown chemicals, mixtures, or metabolites. In contrast, for assays assessing specific and well-characterized mechanisms, such as estrogen receptor (ER) activity, a small number of potent chemicals often account for the majority of the observed effects.^13,14^

In recent years, the application of EBMs in combination with targeted analysis has been strongly recommended for water quality assessment and monitoring with the aim of providing a more robust output for risk assessment and management.^15^ Although EBMs are not yet officially implemented as regulatory tools for drinking water quality monitoring, the continuously increasing number of studies using EBMs to assess the quality of diverse water sources highlights their potential. For instance, the recent regulatory approval of the application of ER and AhR bioassays for monitoring the constituents of emerging concern in recycled water intended for indirect and nonpotable reuse in several facilities in California^16,17^ displays a promising panorama for further regulatory acceptance of EBMs. Moreover, The latest proposal of the Commission of the European Union to incorporate compulsory effect-based monitoring for surface water for all member states^18^ and the acknowledged potential of EBMs for catchment-to-consumer monitoring mentioned in the Australian Drinking Water Guidelines,^19^ present a favorable prospect for regulatory acceptance and eventual mandatory incorporation of EBMs in the United States, the European Union, Australia, and potentially other countries already applying these bioanalytical tools.

While EBMs are used in several countries, there are still geographical areas where there are no known reports of the application of EBMs for water quality assessment. One of those countries is Mexico, where water quality assessment for raw and treated water solely relies on targeted analysis.^20^ Mexico City and its surrounding states face significant challenges related to water scarcity and pollution as freshwater sources used to produce potable water (24% Cutzamala system and 68% aquifers) are under extreme pressure due to overexploitation.^21^ The Cutzamala system serves as treatment and distribution network supplying with water to approximately 6 million people in Mexico City and the State of Mexico.^20^ Water quality before and after conventional treatment meets the regulatory standards according to the Mexican Official Standards (NOM by its acronym in Spanish). For instance, the NOM-001-SEMARNAT-2021, which is regulated by the Secretariat of Environment and Natural Resources (SEMARNAT by its acronym in Spanish), establishes the maximum permissible limits for pollutants in treated wastewater discharges into aquatic bodies.^22^ Complementary to the previous regulation, the NOM-127-SSA1-2021, which is regulated by the Secretariat of Health (SSA by its acronym in Spanish), establishes the permissible quality standards that treated potable water must meet for direct human use. Moreover NOM-127-SSA1-2021 states that water with origins from the wastewater treatment process cannot be considered as potable water.^23^ However, many people avoid consuming tap water due to perceived concerns of contamination, either from the treatment source or through the distribution system,^24^ leading to reduced public confidence in the quality and safety of the water.

The aim of this study is to use a battery of EBMs to assess the quality and potential presence of hazardous chemical pollutants of bottled water, tap water, and purified drinking water from household filters from three demarcations in Mexico City and two municipalities in the State of Mexico and Morelos states. The findings will be compared to bioanalytical equivalent concentration (BEQ) reports for drinking water from other countries. Moreover, the data will be compared with existing derived human-effect-based trigger (EBT) values and regulatory health guidance values.

## 2 Materials and Methods

### 2.1 Chemicals and Solvents

A detailed description of the chemicals and solvents that were used through the assessments performed in this study is presented in Table S1 in the Supporting Information (Section 1.1).

### 2.2 Water Sampling and Extraction

Water samples from Mexico City and the surrounding states were collected during November and December 2024. Samples were obtained from three different brands of bottled water, tap water from three demarcations in Mexico City, drinking water from two household filters (different brands) that use tap water as their source, tap water from two municipalities in State of Mexico and Morelos state, and tap water from Uppsala in Sweden which served as a reference sample for direct comparison. It is important to point out that in Mexico, tap water, also referred to as potable water, is generally not consumed as drinking water due to perceived concerns from the public regarding cleanliness, quality, and safety. As a result, the main source of drinking water for more than two-thirds of the population comes from different forms of bottled water.^25^ A detailed explanation and description of the samples and sampling strategy are listed in Table 1.

All samples were collected in sterile polystyrene sampling bottles (VWR, cat no. VWRI331-0269), which have previously shown not to interfere or alter with the activities detected in bioassays.^26^ Samples were then stored at 4 °C prior to transportation. The samples were transported by air from Mexico to Sweden under controlled conditions, with a total travel time of approximately 16 h. Upon arrival to the laboratory for processing, samples were extracted using solid-phase extraction (SPE), then eluted with 99% ethanol, and evaporated until the samples were enriched 5000 times and later stored at −20 °C until the effect-based assessment. The water sample concentrations were expressed as the relative enrichment factor (REF) and were diluted 100 times in the cell culture medium. The resulting highest REF concentration for all samples (REF 50) was calculated by dividing the enrichment factor of the SPE by the dilution factor for the *in vitro* assays. Additional REF concentrations (25, 12.5, and 6.25) were prepared by performing serial dilutions departing from REF 50. A more detailed description of sample preparation and extraction is included in the Supporting Information (Section 1.2).

### 2.3 Effect-Based Assessment

All water samples were assessed with a battery of EBM’s representing important toxicity pathways of human health relevance and which also have been linked to display bioactivity in response to known and unknown chemical pollutants in water. These pathways encompass nonspecific modes of action such as cytotoxicity as well as specific modes of action such as endocrine disruption (ER and androgen receptors [AR] activation/inhibition), genotoxicity (micronuclei formation [MN+]), activation of the AhR, and translocation of the Nrf2 transcription factor indicative of oxidative stress. A more detailed description of the methods used in this study is shown in Table 2. In addition, the selection criteria for the bioassays used in this study were based on the availability of OECD test guidelines, the frequency of use, and validation on several scientific reports. For the specific case of the novel ER-isjaki assay, this one was preferred over other existing assays due to its high sensitivity to detect estrogenic activity.^29^ Additional information on cell culture, maintenance, and bioactivity testing is presented in the Supporting Information (Sections 1.3 and 1.5)

All assays, except for genotoxicity assessment which is carried out using flow cytometry, are reporter-gene-based assays for which water sample bioactivity detection was measured by luminescence readings. Moreover, to generate assay-specific dose–response curves, required to obtain effect concentration (EC) or inhibitory concentration (IC) values for each bioassay, that in turn are needed to calculate BEQ values, assay-specific reference compounds (see Table 2) were run together with water samples, vehicle control, and DMSO 10% or 15% as cytotoxicity positive control. The final concentration of ethanol for vehicle control, water samples, and reference compounds was set at 1% for all bioassays. Finally, cell viability was assessed in parallel for each *in vitro* test (EMA+ for genotoxicity and MTS for all other assays) with the aim of ensuring that no cytotoxic effect would mask any biological effects, thus guaranteeing that the obtained data is end point-specific and reliable. Additional details on cell viability testing are presented in the Supporting Information (Section 1.4).

### 2.4 Data Analysis

To ensure the quality and reliability of the data generated from the effect-based assessment, cell viability was screened for all samples. In this case, any concentrations inducing cytotoxicity (cell viability below the cutoff limits presented in Table 2) were excluded for EC or IC value calculations.

Bioactivity assessment for agonistic reporter-gene assays (AhR, AR, and ER) was done by normalizing luminescence data to the average of vehicle control, then by standardizing to zero, removing the vehicle control average, and finally by converting data into % of assay maximum (set to 100%) by normalizing with the highest concentration of the reference compound. For AR antagonistic effects, data were normalized to dihydrotestosterone (DHT)-spiked vehicle control and then converted into % of the assay maximum. For Nrf2 activity and micronuclei (MN+/EMA+) assay, data were normalized to vehicle control and expressed as fold change. Table 2 presents the effect level (%) defined as the cutoff values (EC or IC) for each bioassay (except for MN+) used to determine samples’ bioactivities, and the Supporting Information, Figure S1, shows the dose–response curves for the reference compound of each bioassay. For bioactive samples and reference compounds, the normalized data were used to calculate EC_10_, EC_20_, EC_IR1.5_, or IC_30_, which represent the sample concentration expressed as REF required to induce a specific biological activity. In the case of MN+, noncytotoxic samples inducing micronuclei formation equal or higher than 3-fold were labeled as genotoxic. GraphPad Prism (v. 10.5.0) was the software used to generate concentration–response curves for reference compounds, effect-specific, and cell viability bar plots as well as to determine the EC or IC values for each assay and to calculate the EC or IC for bioactive samples.

To calculate the BEQ values for bioactive samples, the EC or IC of the reference compound was divided by the EC or IC or the sample as described in the following equation,^30,31^ where EC*_x_* represents the effect level for each assay

$$\mathrm{BEQ}=\frac{{\mathrm{EC}}_{x}\;\mathrm{reference}\;\mathrm{compound}}{{\mathrm{EC}}_{x}\;\mathrm{sample}}$$

All obtained BEQ values are presented in Table 3 and Section 3, and the average limit of detection (LOD) was calculated using the EC of the reference compound and the REF concentrations. These data are presented in the Supporting Information, Table S2. The mean and standard deviation (SD) of all experiments presented in this study were calculated from all technical replicates per concentration from all independent experiments.

**Table 1:** Water Sample ID, Description, Location, and Specifications

sample ID	sample description/location	specifications
water jug	bottled water in a water jug from a major brand	a water jug is a 20 L container made of thick plastic in which treated groundwater serves as primary drinking water source for around 76% of the Mexican population.^27^ Water jugs are usually washed, reused several times, and transported under different conditions and temperatures
brand #1	bottled water from brand #1	treated groundwater in standard 1 L plastic containers
brand #2	bottled water from brand #2	
BJ tap water	tap water from Benito Juárez demarcation in Mexico City	water is supplied by a mixture of sources including the Cutzamala system and local aquifers stored in wells^20^
Iz tap water	tap water from Iztapalapa demarcation in Mexico City	
filter brand #1 BJ water	purified water from household water filter brand #1 that uses Benito Juárez demarcation tap water as its source	filtration system steps according to manufacturer:
		(1) microfiber filtration
		(2) activated carbon filtration (carbon trap)
		(3) controlled-release chlorine compound device
		(4) activated carbon filtration (carbon polisher)
		(5) ultrafiltration
		the filter has been in continuous use for approximately 5 years.
filter brand #2 Iz water	purified water from household water filter brand #2 that uses Iztapalapa demarcation tap water as its source	filtration system steps according to manufacturer:
		(1) ceramic filtration
		(2) activated carbon filtration
		(3) silica sand filtration
		(4) water storage on mineral stones impregnated with silver to avoid bacterial growth and maintain pH
		the filter has been in continuous use for approximately 2 years.
Az	tap water from Azcapotzalco demarcation in Mexico City	water is supplied by a mixture of sources including the Cutzamala system and local aquifers stored in wells^20^
EdoMex	tap water from Neza municipality in the State of Mexico (Estado de México in Spanish)	
Mor	tap water from Cuautla municipality in the Morelos state	water is directly supplied from the local aquifer and corresponding treatment plant
Uppsala	twater from the city of Uppsala in Sweden	water is directly supplied from groundwater from Uppsalaåsen esker and corresponding treatment plant^28^

**Table 2:** Summary of *In Vitro* Effect-Based Tools Used with Corresponding Reference Compound, Effect Concentration Levels, and Their Experimentally Measured Range for Eventual BEQ Calculations^a^,^b^,^c^,^d^,

toxicological end point	cell line	reference compound	effect level (%) to define BEQ	EC/IC range^c^	cytotoxicity test and cytotoxic cutoff	reference document
oxidative stress (Nrf2 activation)^a^	MCF-7 AREc32	*tert*-butylhydroquinone (tBHQ)	EC_IR1.5_	3.1–3.3 μM	MTS/≤80% of vehicle control	
androgen receptor activation	AR-EcoScreen GR-KO M1	dihydrotestosterone (DHT)	EC_20_	23–32 pM		OECD 458^d^ ^,^ ^32^
androgen inhibition		hydroxyflutamide (OHF)	IC_30_	12–27 nM		
aryl hydrocarbon receptor activation	DR-EcoScreen	2,3,7,8-tetrachlorodibenzo-p-dioxin (TCDD)	EC_10_	1.8–2.3 pM		
estrogen receptor activation	ER-isjaki assay (MCF-7 transiently transfected with *pnlERE[secNluc/Hygro]* plasmid)	17β-estradiol (E2)	EC_20_	0.38–0.47 pM		^29^
*in vitro* micronuclei formation (genotoxicity)	TK6	mitomycin C	N.A.^b^	N.A.	EMA+/≤ 4-fold change of vehicle control	OECD 487^d^ ^,^ ^33^

a Data are expressed as fold change (normalized to vehicle control) for the Nrf2 activation bioassay.

b For genotoxicity, no BEQ is calculated; instead, samples are labeled as genotoxic or nongenotoxic based on a ≥3-fold induction when compared with the vehicle control.

c EC/IC range calculated from the dose–response curves performed for this study.

d Referenced *in vitro* assays were performed according to international guideline documents but with minor modifications. A detailed description of the experimental procedures is given in the Sections 1.3 and 1.5.

N.A (Not applicable)

**Table 3:** BEQs of Bioactive Water Samples for AhR Activity, AR Inhibition, and ER Activation. <LOD indicates samples that were below the LOD and therefore considered non bioactive

		bioanalytical equivalents		
water category	water sample	AhR activation TCDDeq	AR inhibition OHFeq	ER activation E2eq
		average BEQ (pg TCDDeq/L) ± SD	average BEQ (ng OHFeq/L) ± SD	average BEQ (pg E2eq/L) ± SD
bottled water	water jug	<LOD	<LOD	26 ± 14
	brand #1			6 ± 3
	brand #2			7 ± 3
tap water vs household-filtered water	BJ tap water	27 ± 6	<LOD	3 ± 0.01
	filter #1 BJ tap water	<LOD	<LOD	<LOD
	Iz tap water	27 ± 2	141 ± 17	
	filter #2 Iz tap water	<LOD	106 ± 24	
tap water	Azcapotzalco	<LOD	111 ± 55	13 ± 3
	State of Mexico		<LOD	7 ± 0.1
	Morelos			6 ± 5
	Uppsala, Sweden			<LOD

## 3 Results and Discussion

### 3.1 Bioactivities

#### 3.1.1 Cell Viability

No cytotoxicity was detected for any of the water samples in any of the different cell lines used for effect-based assessment in this study (MCF-7 AREc32, TK6, DR-EcoScreen, AR-EcoScreen, and MCF-7 transfected with the *pNL2.3-ERE­[secNluc/Hygro]* plasmid), as none of the samples were below the cell viability cutoff limit (≤80%). As a result, all concentrations for all samples were included for analysis (Supporting Information, Figures S2–S6).

#### 3.1.2 Oxidative Stress and Genotoxicity

In the case of Nrf2 activation, which is a signaling pathway involved in antioxidant defense in the presence of reactive oxygen species (ROS) and oxidative stress, none of the samples were bioactive. However, one of the samples, tap water from Iztapalapa demarcations at the highest concentration, was close to the cutoff limit (≥1.5-fold change) (Supporting Information, Figure S7). With regard to genotoxicity, which induces DNA damage potentially leading to genetic mutations, and to oxidative stress, which in some cases has been linked to the formation of multinucleated cells, none of the tested samples induced MN+ (≥3-fold change) (Supporting Information; Figure S8), which is consistent with our findings on Nrf2 activity. Although NOM-127-SSA1-2021 describes chlorination (0.2 – 1.5 mg/L free residual chlorine) as the main disinfection process during water treatment,^23^ these findings indicate the absence of compounds or disinfection byproducts directly targeting DNA or inducing ROS formation which in turn triggers oxidative stress and genotoxicity, or that such compounds are at very low concentrations. Moreover, the lack of bioactivity in all tested samples is a positive indicator of the water quality, as studies in outgoing treated drinking water from Korea^34^ and tap water from Spain^35^ have reported bioactivities related to oxidative stress. In addition, another study in a Swedish drinking water treatment plant (DWTP) reported oxidative stress and micronuclei formation in the raw water and occasionally in the outgoing water and throughout the distribution system.^3^

#### 3.1.3 Aryl Hydrocarbon Receptor Activity

For the assessment of AhR activity, which modulates immune regulation and metabolism of environmental pollutants, only 2 of the samples were bioactive (Figure 1b), while the remaining samples were below the cutoff limit (≥10% effect) (Figure 1). In this case, tap water from Benito Juárez and Iztapalapa demarcations displayed bioactivity at REF 50 where both samples showed an activity of around 17% of the max effect and was slightly bioactive at REF 25 where an approximate 10% of max effect was reached for both samples. The calculated BEQs for both samples were at 27 pg TCDDeq/L (Table 3). When AhR activities from tap water are compared with those of the outgoing water from household filters, there is an indication that both filter brands are efficiently removing pollutants that trigger activation of AhR below the LOD (Figure 1b). By comparing our effect-based data with other existing AhR activity data reported for drinking water, it can be appreciated that the BEQ values for tap water are within the range of values reported in other studies and in some cases below these values. For instance, a study at a DWTP in Australia reported an average bioactivity of 170 pg TCDDeq/L in finished treated water.^36^ Another study carried out at a DWTP in Stockholm, Sweden measured AhR activity ranging from 0.8 to 198 pg TCDDeq/L in water from the distribution system.^3^ An additional study assessing AhR activity in outlet water at another Swedish DWTP and tap water from the distribution network in Uppsala detected low bioactivity levels which were slightly above the LOD (11 pg TCDDeq/L).^37^ In our study, the tap water sample from Uppsala, which is a mix of waters from the aforementioned DWTP and distribution system, was not bioactive. Although the previous study^37^ reported bioactivities, because these were close to the LOD and even below our calculated LOD (13 pg TCDDeq/L, see Supporting Information, Table S2), it can be stated that there are no relevant differences in the presence of AhR activity in tap water between both studies.

**Figure 1:**
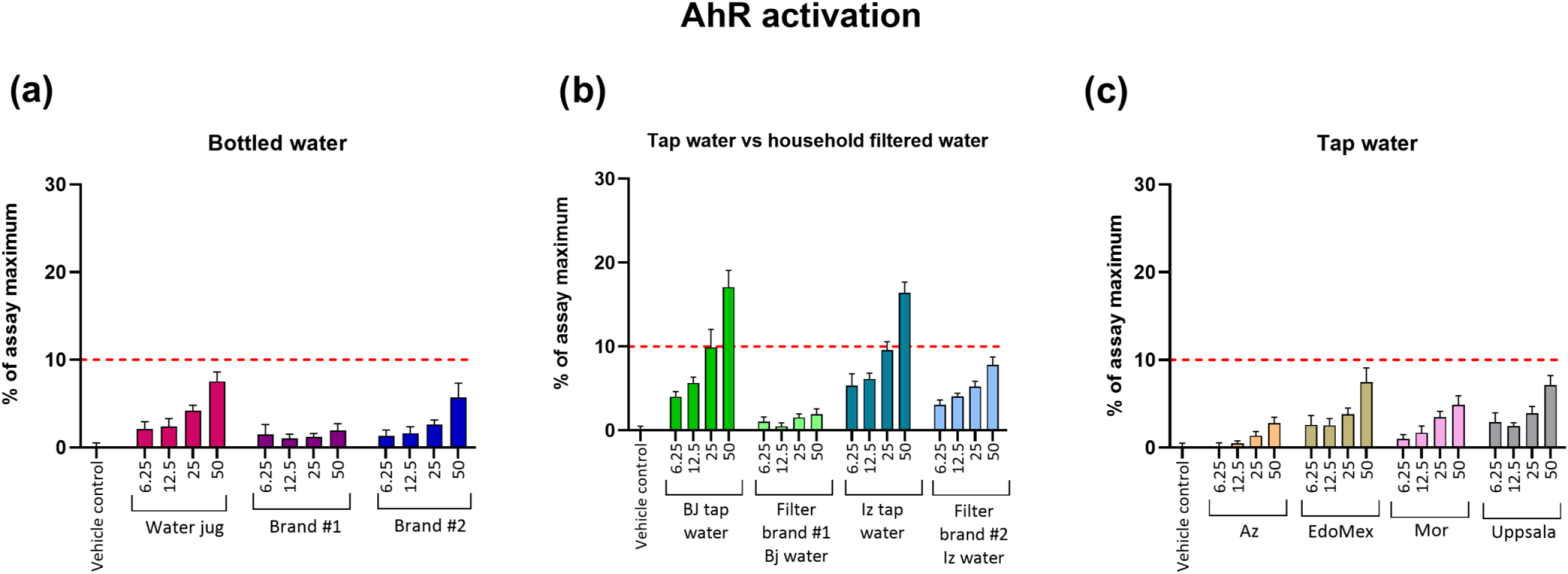
Activity of the AhR in DR-EcoScreen cells exposed to water samples for 24 h. Samples were categorized as (a) bottled water (water jug, brands #1 and #2); (b) tap water and filtered tap water using two different household filter brands in two different demarcations in Mexico City (Benito Juárez and Iztapalapa); and (c) tap water from different sources (Azcapotzalco demarcation in Mexico City, State of Mexico and Morelos states and reference sample from Uppsala, Sweden). Sample concentrations are expressed as the REF (6.25–50). Data are presented as mean ± SD, *n* = 8 (two independent experiments with four technical replicates per concentration), and the dotted line represents the bioactivity cutoff limit in % of assay maximum (10% effect).

The presence of AhR activity in surface water used to produce drinking water and finished drinking water (conventional treatment and granulated activated carbon filtration) as described above, has been continuously detected in several regions of the world.^1,38,39^ Such activity has generally been attributed to a potential mixture of dioxins, pharmaceuticals, polycyclic aromatic hydrocarbons, polychlorinated biphenyls, and other chemicals;^40^ however, such chemicals generally explain less than 10% of the observed biological effects.^41^ For this reason, human EBTs for drinking water have been proposed as a reference screening tool to identify potential concerns in bioactive samples. In this case, a proposed human EBT for AhR activity in drinking water of 60 pg TCDDeq/L^42^ shows that although AhR activity was detected in some of the samples, the BEQ values do not indicate a trigger exceedance under effect-based monitoring. Moreover, when comparing our data with regulatory guidance values such as the maximum contaminant level for TCDD given by the U.S. EPA of 30 pg/L in drinking water,^43^ it can be determined that the BEQ values do not exceed the risk-based screening limit provided by this agency. However, it should be noted that there is currently no scientific consensus on how EBTs should be derived, and it is especially challenging, if not impossible, to derive EBTs for end points, such as AhR, where the activity is caused by many different known and unknown compounds with a high level of diversity in toxicokinetic and toxicodynamic mechanisms. Moreover, EBTs are cell line-specific, and the abovementioned EBT is derived for another cell line than that used in this study. Hence, the comparison toward EBTs or maximum contaminant levels for TCDD should be made with great caution.

#### 3.1.4 Androgen Receptor Activity

For AR activation, which is a nuclear hormone receptor regulating effects of androgens mainly on male development and reproduction, none of the samples displayed bioactivity (≥20% effect) (Supporting Information, Figure S9). In general, the detection of AR agonism in drinking water is usually below the LOD, or if detected, it is below the proposed human EBTs (4.5–32 ng DHTeq/L^6,44^ ). Regarding our results, the lack of bioactivity is in line with several other studies around the world where AR activity is minimal or nondetected,^7,34,45,46^ suggesting that the presence of hormones or compounds activating the AR receptor are either absent or at very low concentration, which is a positive indicator on the water quality of the tested samples.

On the other hand, AR inhibition (≤30% inhibition) was detected in 3 of the samples but only at the highest concentration. Tap water from the Iztapalapa demarcation displayed the greatest inhibition (40% of inhibition effect) (Figure 2b), followed by outgoing water from household filter #2 from the Iztapalapa demarcation (32% of inhibition effect) (Figure 2b) and tap water from the Azcapotzalco demarcation (31% of inhibition effect) (Figure 2c). The calculated BEQs were 141, 106, and 111 ng of OHFeq/L (Table 3). Despite that water from household filter #2 displayed antiandrogenic effects, when compared with tap water from Iztapalapa, we could say that filter #2 is able to reduce the antiandrogenic effect by 8% but does not completely remove the bioactivity. Although the number of studies on antiandrogenic effects, especially in drinking water, is low in comparison with androgenic or estrogenic agonistic effects,^47^ the relevance of using effect-based assessment to detect antiandrogenic effects has gained more attention. This is because known antiandrogenic compounds have been reported to act as potential endocrine disruptors resulting in the alteration of the normal function of androgen hormones.^48^ Regarding the calculated BEQ values, these are generally lower than those reported in other studies. For example, a study carried out at several DWTPs in Sweden, found 847 ng OHFeq/L for outlet finished drinking water at one of the plants.^7^

**Figure 2:**
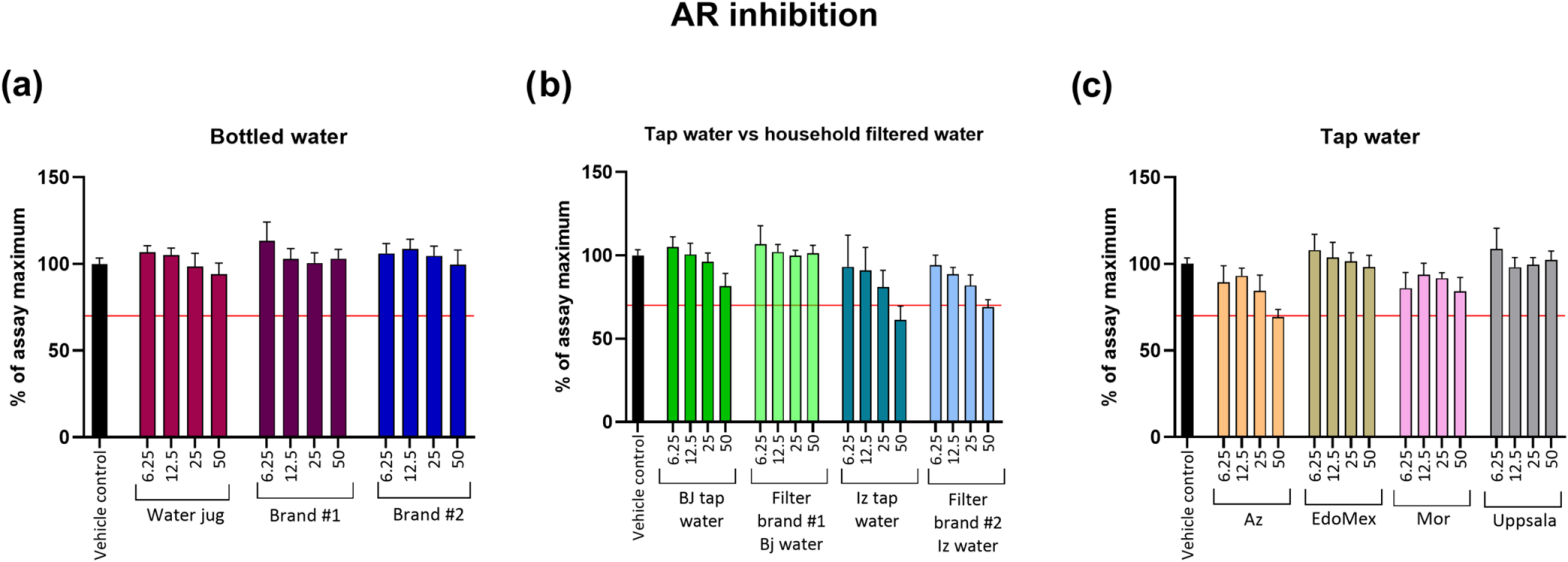
Inhibition of the AR in AR-EcoScreen cells exposed to water samples for 24 h. Samples were categorized as (a) bottled water (water jug, brands #1 and #2); (b) tap water and filtered tap water using two different household filter brands in two different demarcations in Mexico City (Benito Juárez and Iztapalapa); and (c) tap water from different sources (Azcapotzalco demarcation in Mexico City, State of Mexico, and Morelos states and reference sample from Uppsala, Sweden). Sample concentrations are expressed as REF (6.25–50). Data are presented as mean ± SD, *n* = 8 (two independent experiments with four technical replicates per concentration), and the line represents the inhibitory cutoff limit in % of assay maximum (30% inhibition).

However, to make a direct comparison between our BEQ data and the range of proposed human EBTs for antiandrogenic activity of 3300–14,400 ng FLUeq/L,^6,44^ we need to translate flutamide equivalents (FLUeq) into hydroxyflutamide equivalents (OHFeq). To make this translation, FLU’s relative effect potency (REP) is determined first. For this, the IC_50_ values for FLU and OHF are established. For FLU, a literature search indicates that the average IC_50_ in the AR-EcoScreen cell line is 1020 nM,^49^ whereas for OHF, the average IC_50_ was directly calculated from our dose–response curves (77 nM). Next, FLU REP was calculated by dividing OHF IC_50_ by FLU IC_50_, where OHF REP was set to 1 and FLU REP was calculated at 0.075. To translate FLUeq into OHFeq, OHF’s potency ratio of the OHF was calculated. In this case, the OHF REP was divided by the FLU REP, which resulted in the OHF being around 13 times more potent than FLU. Such a potency ratio can be categorized as 1 equiv of OHF equals 13 FLUeq. Based on this calculation, we then obtain an equivalent range of 254–1108 ng of OHFeq/L, which indicates that the BEQs in this study (106–141 ng of OHFeq/L) are lower than the translated range defined by the proposed EBTs. Given that AR inhibition is caused by a wide variety of known and unknown chemicals, and the proposed EBTs were derived from different cell lines, it is important to recognize that the same challenges in deriving AhR EBTs also apply to the derivation of EBTs for antiandrogenic activity.

#### 3.1.5 Estrogen Receptor Activity

For ER activation, which is also a nuclear hormone receptor involved in female reproduction and which is modulated by hormones such as estradiol (E2), 7 samples were determined to be bioactive (≥20% effect). In the case of bottled water samples, water from the jug container displayed the highest bioactivity with 78% of the maximum effect at REF 50. ER activity above the assay’s cutoff limit (20% effect) was still observed at the lowest tested concentration (REF 6.25) with 26% of the max effect (Figure 3a). The BEQ calculated for this sample was 26 pg of E2eq/L (Table 3). Samples from bottled water brands #1 and #2 showed bioactivity levels of 30% and 25% of the maximum effect at the highest concentration (Figure 3a) and their calculated BEQs were 6 and 7 pg E2eq/L, respectively (Table 3). With regard to tap water, most of the samples analyzed were bioactive. The calculated BEQ for tap water from Benito Juárez demarcation (28% of the maximum effect) was 3 pg E2eq/L (Figure 3b and Table 3), while the BEQs for Azcapotzalco demarcation (29% of the maximum effect), State of Mexico, and Morelos states (36% and 28% of the maximum effect) (Figure 3c) were 13, 7, and 6 pg E2eq/L, respectively (Table 3).

**Figure 3:**
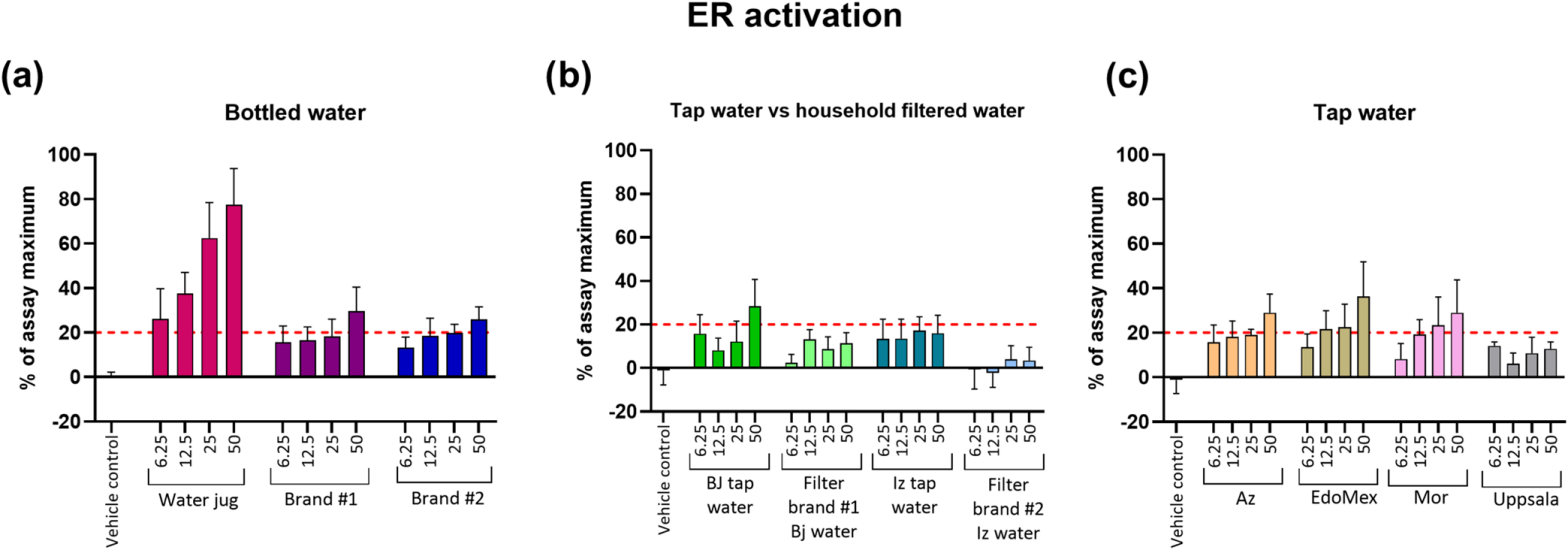
Activity of the ER in MCF-7 cells transiently transfected with 5 ng per well of the *pNL2.3-ERE* plasmid (ER-isjaki assay) and exposed to water samples for 24 h. Samples were categorized as (a) bottled water (water jug, brands #1 and #2); (b) tap water and filtered tap water using two different household filter brands in two different demarcations in Mexico City (Benito Juárez and Iztapalapa); and (c) tap water from different sources (Azcapotzalco demarcation in Mexico City, State of Mexico, and Morelos states and reference sample from Uppsala, Sweden). Sample concentrations are expressed as REF (6.25–50). Data are presented as mean ± SD, *n* = 9 (two independent experiments with three or six technical replicates per concentration), and the dotted line represents the bioactivity cutoff limit in % of assay maximum (20% effect).

The detection of estrogenic activity in water, especially in surface water used to produce drinking water, is a common occurrence. A recent study assessing the water quality of a river in Brazil which serves as the main source to produce drinking water in Rio de Janeiro detected a maximum BEQ concentration equivalent to 5.3 ng E2eq/L.^39^ In terms of water treatment, conventional DWTPs are usually not designed to fully remove estrogens such as E2 and other estrogenic compounds such as ethinyl estradiol.^50^ However, the concentration of estrogens and estrogenic compounds present in the finished treated water is influenced by several factors such as contamination from wastewater effluents into the freshwater source^51^ or the presence of natural estrogens and/or estrogenic compounds in concentrations ranging from pg/L to ng/L.^52,53^

In this study, the estrogenicity values detected for tap water were, in general, lower than the reported values from various studies. For instance, finished drinking water from a DWTP in Iran detected 420 pg E2eq/L,^46^ while another study at a DWTP in Korea measured an average of 38 pg E2eq/L.^34^ A study at a Swedish DWTP reported estrogenicity up to 79 pg E2eq/L.^3^ A more comprehensive study involving several European countries including Australia and South Africa found ER activity ranging from 1 to 80 pg E2eq/L.^45,54^ Regarding estrogenicity found in bottled water, studies assessing the effects of endocrine disruptors from plasticizers in bottled water reported ER activity in ranges from 2 up to 14 pg E2eq/L,^55–57^ which is in line with our data.

Although most of the analyzed samples were bioactive, when comparing the calculated BEQs with the proposed human EBT for estrogenicity (200 pg E2eq/L^6,44^ ) and the WHO’s benchmark value for E2 in drinking water (1000 pg E2/L^58^), our findings indicate that the levels detected for ER were substantially lower than the previously mentioned effect-based reference and human safety threshold. Thus, we can conclude that the estrogenic presence in all bioactive samples does not affect the quality of the water and is of no concern for human consumption.

In this study, we successfully implemented for the first time the ER-isjaki assay to assess estrogenicity in water samples with SPE preconcentration. The ER-isjaki assay is a newly developed assay which in its initial phase using transiently transfected cells has proven to be 10 to 100 times more sensitive than other existing assays. One of the main objectives for such a sensitive assay is to assess very low concentrations of estrogens in surface water with or without requiring sample preconcentration using SPE.^29^ Since estrogenicity was detected in most of the preconcentrated samples, and in some of the cases, the detected concentrations were as low as 3 pg E2eq/L, which was slightly above the calculated LOD of 2 pg E2eq/L at REF 50 for this study (see Supporting Information, Table S2), we could conclude that due to the high sensitivity of this assay, we were able to measure very low concentrations of estrogens that probably other assays would not have detected.

### 3.2 Use of In Vitro Assays to Monitor Water and Water Quality in Mexico

In recent years the use of EBMs as a complementary tool not only to assess water quality, but to routinely monitor the presence of potentially hazardous chemical mixtures in water sources used to produce drinking water, during the treatment process and of finished drinking water has become increasingly popular in several European countries and Australia.^10,15^ After conducting a comprehensive literature search on the application of EBMs for water quality assessment in Mexico, and although an update to the NOM-001-SEMARNAT-2021 included assessment of acute toxicity of treated wastewater using the *Vibrio fischeri* assay,^22^ we did not identify any published studies employing reporter-gene bioassays. It is important to highlight that data from the ecotoxicological *V. fischeri* assay cannot be directly compared with the effect-based methods used in this study as that bacterial test measures toxicity in a nonpathway specific manner and is sensitive to high concentrations of toxicants but not to specific classes of chemicals at low concentrations.^59^ Moreover, no reports of the application of EBMs in Mexico for freshwater and drinking water quality assessment or removal efficiency at DWTPs were found. This lack of studies highlights a research gap, especially considering that EBMs are increasingly recognized internationally as complementary tools for monitoring complex chemical mixtures and assessing treatment performance. For this reason, we developed a study using a battery of bioanalytical tools for oxidative stress, genotoxicity, AhR activation, AR agonism/antagonism, and ER activation to assess the quality and possible presence of hazardous pollutants of different potable and drinking water sources in Mexico City and surrounding states.

Based on our findings primarily supported by comparisons with other international studies, proposed human EBTs, and to a lesser extent with regulatory health guidance values for drinking water, we consider that there is no clear indication of any potential concerns for the quality of sampled bottled, tap, and household-filtered waters. Supporting this conclusion, our data showed that BEQs for bioactive samples were (a) below or within the same range of reported values from other studies worldwide, (b) below the proposed EBTs for drinking water, (c) below health guidance values where direct comparisons were possible, and (d) comparable with the findings on water from Uppsala, Sweden. It is crucial to mention that in the present study, estrogenicity and genotoxicity end points serve as strong indicators of the absence or low levels of hazardous chemicals in water, thus reflecting good water quality. This is supported by the measured estrogenic BEQs below the EBT and regulatory guidance value and the absence of genotoxic activity (negative MN+) across all samples, which suggest that adverse effects are unlikely to be triggered, hence, not posing a risk to human health. However, in the case of Nrf2, AhR, and AR antagonism end points, although some of the samples were non-bioactive and none of the bioactive samples exceeded the proposed EBTs, it is more complex to draw conclusions on water safety. This is because biological activities on the abovementioned end points are triggered by a wide variety of known and unknown chemicals. As a result, EBTs are not yet fully established, as these are based on single reference compounds representing only some of the most relevant and well-known compounds that trigger specific modes of action. Therefore, concluding on potential risks from chemical mixtures triggering biological activities should be done with caution.

It is also worth noting that, although the detected levels of bioactivities do not pose an immediate concern to water quality, the presence of heavy metals in water in several areas of Mexico City has been documented.^60^ Heavy metals such as arsenic, lead, iron, manganese, and mercury have been detected at concentrations above the maximum permissible limits established by the NOM-127-SSA1-2021 regulation. These exceedances have been detected in several aquifers and on the distribution networks that supply tap water, particularly affecting regions within the Iztapalapa demarcation, with reports dating back to 2017.^60^ In this case, the SPE cartridges used for the sample extraction process are not designed to retain free heavy-metal ions, as these are designed based on a hydrophilic–lipophilic balanced copolymer for capturing organic analytes. As a result, if heavy metals were present in the tap water of the Iztapalapa demarcation or in any other of the tested samples, their adverse effects may not have been captured by the bioassays. Therefore, further assessments of samples, especially from the Iztapalapa demarcation, without the SPE sample concentration are required to evaluate the potential adverse effects associated with the presence of heavy metals.

Finally, when comparing the findings of Mexican water samples with our reference sample from Uppsala, Sweden, we can determine that overall and considering that our BEQ values were below the proposed EBTs, the quality of the assessed Mexican waters is similar to that of the tap water sample in Uppsala City. It is important to note that Mexican water quality data from this study cannot be directly compared with the findings for tap water from Uppsala as this sample was consistently non-bioactive for all end points, whereas several Mexican samples were bioactive for various end points as it has been previously discussed. However, and taking into account the comparison of our BEQs with values from other countries, including Sweden, with EBTs and regulatory health guidance values, we can conclude that in general, all tested samples are of good quality.

## 4 Conclusions

In this study, we used a battery of EBMs to assess the quality and potential presence of hazardous chemical pollutants in bottled water, tap water, and purified drinking water from household filters from three demarcations in Mexico City and two municipalities in the State of Mexico and Morelos states. The findings indicate that there is no clear indication of any potential concerns regarding the quality of any of the samples analyzed. This is because our values were below or within range with the BEQs reports from other countries, were below the proposed human EBTs for all end points, and were below regulatory guidance values where direct comparisons were possible. Hence, it can be concluded that, overall, the assessed drinking and potable water samples are of good quality. Moreover, the study further highlighted the benefits of using EBMs as tools for water quality assessment and as indicators of the presence of potential chemical hazards.

## Supplementary Material



## Notes

Additional experimental details for water extractions, effect-based methods culturing procedures, bioactivity/cell viability assessments, dose–response curves of each bioassay, limits of detection, cell viability graphs for all performed bioassays and bioactivity graphs for Nrf2, genotoxicity and AR bioassays (PDF)
