# Supplementary material for: Effect-Based Assessment of the Quality and Potential Presence of Hazardous Chemical Pollutants in Drinking and Potable Water in Mexico City
Source: ACS ES T Water. 2025 Dec 29;6(1):306–16. doi: 10.1021/acsestwater.5c01058 (PMC12797476; doi:10.1021/acsestwater.5c01058)
Supplement: Supplementary file 1 [file ew5c01058_si_001.pdf]

Supporting Information

Effect-based assessment of the quality and potential presence of hazardous chemical pollutants in drinking and potable water in Mexico City

Aline Colonnello Montero*, Geeta Mandava, Johan Lundqvist

Department of Animal Biosciences, Faculty of Veterinary Medicine and Animal Science, Swedish University of Agricultural Sciences, 756 51 Uppsala, Sweden

Content

1. Supplementary materials and methods

1.1 Chemicals and solvents
1.2 Sample preparation and extraction
1.3 General cell culturing conditions
1.4 Cell viability
1.5 Bioassays activity testing

2. Supplementary figures and tables

Table S1. Chemicals and solvents with supplier, CAS No. and purity
Figure S1. Dose-response curves for the reference compounds of each bioassay
Table S2. Limits of detection
Figure S2. Cell viability of MCF-7 AREc32 cells
Figure S3. Cell viability (EMA+) of TK6 cells
Figure S4. Cell viability of DR-EcoScreen cells
Figure S5. Cell viability of AR-EcoScreen cells
Figure S6. Cell viability on the ER-isjaki assay (MCF-7 transiently transfected with *pNL2.3-ERE* plasmid)
Figure S7. Nrf2 activation
Figure S8. Genotoxicity
Figure S9. AR activity

Corresponding Author

***Aline Colonnello Montero** - Swedish University of Agricultural Sciences, Department of Animal Biosciences, Uppsala, Sweden; orcid.org/0009-0001-8813-4833; Email: aline.colonnello.montero@slu.se

1. Supplementary materials and methods

1.1 Chemicals and solvents

Table S1. Chemicals and solvents with supplier, CAS No. and purity

Compound	Supplier	CAS No.	Purity
tert-Butylhydroquinone (tBHQ)	Sigma Aldrich	1948-33-0	97%
Dihydrotestosterone (DHT)		521-18-6	≥97.5%
Hydroxyflutamide (OHF)		52806-53-8	≥98%
2,3,7,8-tetrachlorodibenzo-n p-dioxin (TCDD)		1746-01-6	-
17β-Estradiol (E2)		50-28-2	≥ 98%
Mitomycin C		50-07-7	≥ 98%
Methanol	Solveco Sweden	67-56-1	≥99.8%
Ethanol	Solveco Sweden	64-17-5	≥99.5%
Dimethyl sulfoxide (DMSO)	Sigma Aldrich	67-68-5	≥ 99.9 %

1.2 Sample preparation and extraction

For sample preparation, 900 ml of each water sample were extracted using solid phase extraction (SPE) with the Oasis PRIME HLB 6cc (200 mg) Extraction Cartridges (Waters), using an 8-channel automated SPE system (SPE-03 Gen 4, MOD-00P system, Promochrom Technologies). For the extraction process, the SPE cartridges or columns were prepared by preconditioning them with 5 ml methanol 99% (5 ml/min flow rate), 15 ml ethanol 95% (5 ml/min flow rate) and 10 ml Milli-Q water (5 ml/min flow rate). Next, samples were added with a pre-selected flow rate of 15 ml/min followed by air-purging with 5 ml air (10 ml/min flow rate) to dry the columns. Samples were then eluted with 6 ml ethanol 99% (5 ml/min flow rate) and a final air-purge with 10 ml air (5 ml/min flow rate) to finalize sample collection. For sample concentration, the ethanol was evaporated by vacuum using the SpeedVac SPD120 Vacuum Concentrator (ThermoFischer Scientific) which in turn is connected to the pump/refrigerating system (UVS420 Universal Vacuum System, ThermoFischer Scientific). Samples were evaporated at 45 °C until these were concentrated to a small volume which later was adjusted and resuspended with ethanol 99% to a final volume of 180 µL, resulting in a concentration factor of 5000.

1.3 General cell culturing conditions

Oxidative stress (Nrf2 activation): The stably transfected MCF-7 AREc32 cell line (derived from human breast adenocarcinoma cells) was obtained from Ximbio (London, UK). Cells were cultured in Dulbecco's Modified Eagle Medium (DMEM GlutaMAX) with 4.5 g/L D-glucose (GIBCO), supplemented with 10% fetal bovine serum (FBS, GIBCO), 50 units/ml of penicillin and 50 µg/ml of streptomycin (1% Pen-Strep, GIBCO) and 0.8 mg/mL Geneticin (G418) selective antibiotic (Life Technologies). For the oxidative stress assay, $1.3x10^5$ cell/ml were seeded in white 384 well-plates with transparent bottom (Costar® Corning Incorporated) and for cytotoxicity assessment in transparent 384 well-plates. Cells were seeded in assay medium consisting of the same components as culture medium but without G418.

Androgen receptor (AR) activation/inhibition: The stably transfected AR-EcoScreen GR-KO M1 cell line (derived from CHO cells with GR gene knockout) was obtained from the Japanese Collection of Research Bioresources Cell Bank (JCRB, Osaka, Japan). Cells were cultured in DMEM Nutrient

Mixture F-12 without phenol red (DMEM-F12, GIBCO), supplemented with 10% FBS, 1% Pen-Strep, 1% L-glutamine (GIBCO), 25 µg/ml of Hygromycin B (InvivoGen) and 50 µg/ml of Zeocin (Invitrogen). For AR agonism/antagonism assays, $1x10^5$ cell/ml were seeded in white 384 well-plates with transparent bottom and for cytotoxicity assessment in transparent 384 well-plates. Cells were seeded in assay medium comprised of DMEM F-12 without phenol red, supplemented with 10% dextran-coated charcoal treated (DCC-FBS, GIBCO), 1% Pen-Strep, 1% L-glutamine. In AR antagonism assays, samples were spiked with 200 pM of Dihydrotestosterone (DHT) with the aim of saturating androgen receptors.

Aryl hydrocarbon receptor (AhR) activation: The stably transfected DR-EcoScreen cell line (derived from mouse hepatoma cells) was obtained from JCRB (Osaka, Japan). Cells were cultured in α-Minimum Essential Media (α-MEM, GIBCO), supplemented with 5% FBS, 1% Pen-Strep and 150 µg/ml of Hygromycin B. For AhR activity assays, $1x10^5$ cell/ml were seeded in white 384 well-plates with transparent bottom and for cytotoxicity assessment in transparent 384 well-plates. Cells were seeded in assay medium containing the same components as culture medium without Hygromycin B.

Estrogen receptor (ER) activation (ER-isjaki assay): The MCF-7 cell line was obtained from the American Type Culture Collection (ATCC, Manassas, VA, USA). The general methodological procedure for this assay was carried out as described by Colonnello Montero *et al.*, 2025 [29]. Cells were cultured in DMEM high glucose, without glutamine and phenol red (GIBCO), supplemented with 10% FBS and 1% Pen-Strep. For the ER activity assay and cytotoxicity assessment, $1x10^5$ cell/ml were seeded in transparent 96-well plates in estrogen-free medium (EFM) containing 5% dextran-coated charcoal treated (DCC, GIBCO) and 1% Pen-Strep. Forty-eight hours after seeding, cells were transiently transfected with 5 ng/well of the *pNL2.3-ERE[secNluc/Hygro]* plasmid. After 24 hours, the medium was replaced with fresh EFM, and exposure was carried out.

Genotoxicity: The TK6 cell line (lymphoblastic cells) was obtained from the ATCC (Manassas, VA, USA). Cells were cultured in Roswell Park Memorial Institute (RPMI, GIBCO) 1640 medium with L-glutamine, supplemented with 10% FBS and 1% Pen-Strep. To assess micronuclei formation and cytotoxicity assessment, $4x10^5$ cell/ml were seeded in round-bottomed transparent 96 well-plates and soon after seeding, exposure was carried out.

Cells were maintained in an incubator at 37 ˚C in 5% CO_2. Culture medium was changed every second to third day. Trypsin-EDTA (Gibco) was used for passaging the cells.

All samples were tested for cell viability and proceeded with reporter assays starting at relative enrichment factor 50 (REF 50) in all cell lines. Vehicle controls consist of 1% ethanol, equivalent to the ethanol concentration in the water samples, and positive controls were included. Vehicle controls were tested in 8 replicates while the samples and reference/positive controls were tested in 4 - 6 replicates. The vehicle controls did not cause any compromised cell viability.

1.4 Cell viability

Plated MCF-7 AREc32, AR-EcoScreen GR-KO M1and DR-EcoScreen cell lines were incubated for 24 hours, cells were exposed to vehicle control, water samples and DMSO 10% as positive control for 24 hours. Cytotoxicity was assessed using the MTS cell viability method. For this procedure, 10 µL CellTiter 96 AQueous One Solution Cell Proliferation Assay, Promega were manually added to each well and incubated for 40 minutes at 37 °C. For the ER-isjaki assay, transiently transfected

MCF-7 cells in plates were exposed to vehicle control, water samples and DMSO 15% as positive control. Cell viability was assessed as described above with the difference that 20 µL of MTS reagent were added to each well and later incubated for one hour and thirty minutes at 37 °C. For all MTS cell viability assays, absorbance was measured at 490 nm on the Spark® Multimode Microplate Reader (TECAN, Austria, GmbH).

1.5 Bioassays activity testing

Reporter-gene assays: After 24 hours of plating, MCF-7 AREc32, AR-EcoScreen GR-KO M1, DR-EcoScreen and transiently transfected MCF-7 cells were exposed to vehicle control, water samples and reference compounds for 24 hours. In all bioassays except for ER activity, cells were lysed, and luminescence was measured using the Luciferase Assay System reagents (Promega) and the TECAN Microplate Reader with an automatic syringe injector for addition of the luciferase substrate to each well. For ER activity assays, 10 µl of cell culture medium, 10 µl of Milli-Q water and 10 µl of Nano-Glo® Luciferase Assay reagent (Promega), were added to a white 384-well plate with transparent bottom and luminescence was measured using the TECAN Microplate Reader.

Genotoxicity assessment: After TK6 cells were exposed to vehicle control, water samples and reference compound, micronuclei formation and cell viability were analyzed at the same time by flow cytometry using the FACSVerse 8 color Flow Cytometer (BD BioSciences, Franklin Lakes, NJ, USA) with BD FacSuite v1.0.6 software. Staining was carried out as described by the protocol *In vitro* MicroFlow 96 Well Plate Fromat Kit (Litron Laboratories, NY, USA). Cells were stained with ethidium monoazide or EMA (red dye) to determine cell viability, next cells were lysed, and chromatin was stained with SYTOX Green (green dye). Flow cytometry measurements were conducted by collecting a maximum of 20 000 gates events per sample. The FITC channel measured the green dye fluorescence and PerCP-Cy5.5 channel for EMA the red dye fluorescence.

References:

(29) Colonnello Montero, A.; Mandava, G.; Oskarsson, A.; Lundqvist, J. Development of a highly sensitive reporter gene cell line for detecting estrogenic activity (the ER Isjaki assay). *Environ Int* **2025**, *202*, 109635. DOI: 10.1016/j.envint.2025.109635 From NLM.

2. Supplementary figures and tables

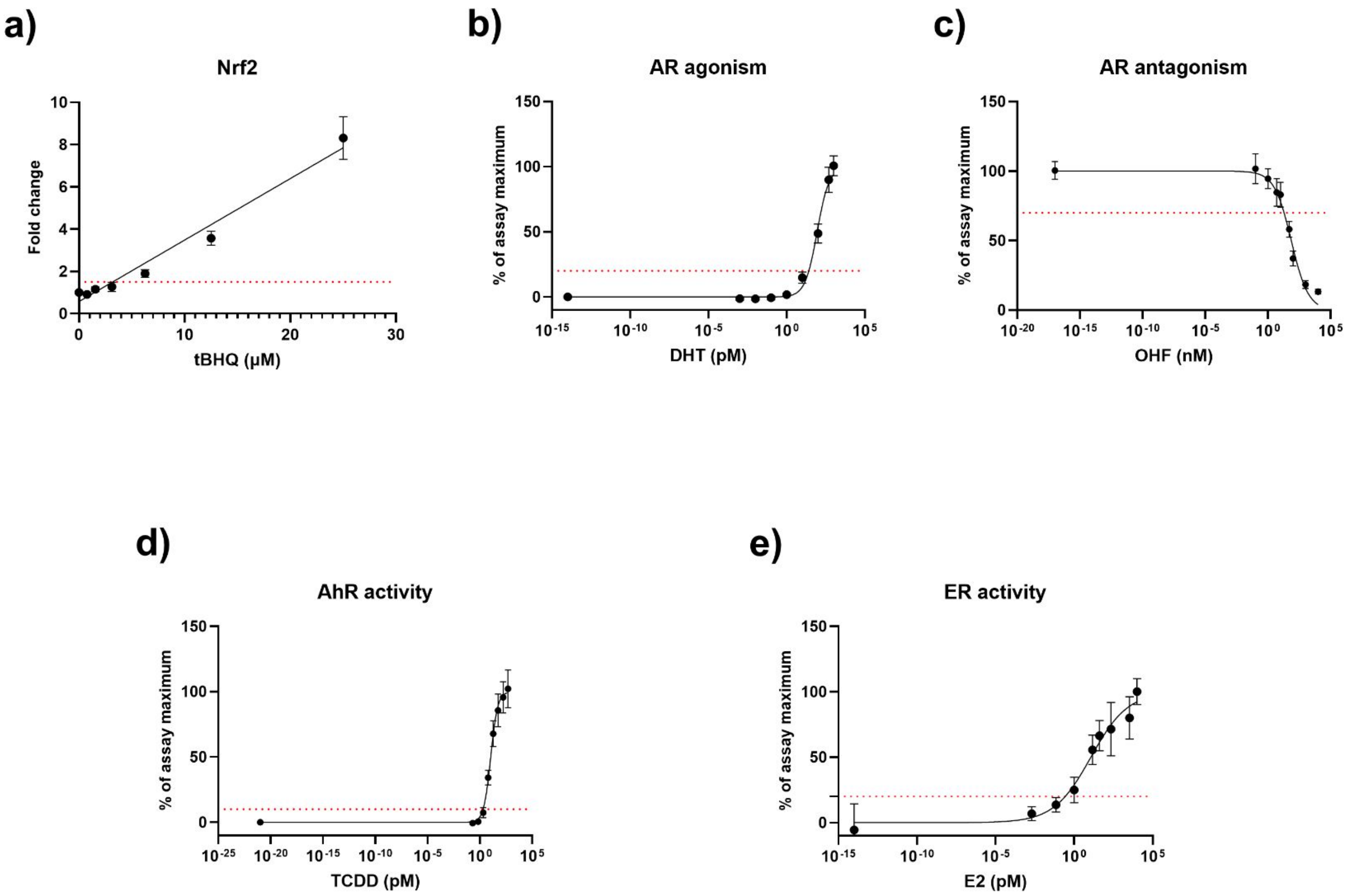

Figure S1. Summary of the dose-response curves for the reference compounds of each performed bioassay. For the oxidative stress induction, **a)** Tert-Butylhydroquinone (tBHQ) curve, for AR induction **b)** Dihydrotestosterone (DHT) curve, for AR inhibition **c)** Hydroxyflutamide (OHF) curve, for AhR activity **d)** 2,3,7,8-tetrachlorodibenzo-p-dioxin (TCDD) curve and for ER induction **e)** 17β-estradiol (E2) curve. Data is presented as mean ± SD, n= 8 (two independent experiments with four technical replicates per concentration), except for ER induction where n= 9 (two independent experiments with three or six technical replicates per concentration). The dotted lines represent the bioactivity cut-off limit in fold change or % of assay maximum for each bioassay (Nrf2 1.5; AR agonism 20%; AR antagonism 30%; AhR activity 10%; ER activity 20%).

Table S2. Limits of detection (LOD) for AR inhibition, AhR and ER activation where water samples were bioactive. Data is presented as average LOD ± SD. For AR inhibition and AhR activation, n= 8 (two independent experiments with four replicates per concentration). For ER activity, n= 9 (two independent experiments with three-six replicates per concentration).

Bioassay	LOD ± SD				
	REF 50	REF 25	REF 12.5	REF 6.25	REF 3.1[1]
AR inhibition (ng OHFeq/L)	99 ± 40	198 ± 83	N.A.	N.A.	N.A.
AhR activation (pg TCDDeq/L)	13 ± 2	26 ± 5	53 ± 9	N.A.	N.A.
ER activation (pg E2eq/L)	2 ± 0.3	5 ± 0.7	9 ± 1	18 ± 3	37 ± 5

[1] This REF concentration was not experimentally tested. However, due to the high sensitivity of our estrogenic activity assay, the detected and quantified ER-activity in pg E2/L equivalents for the most bioactive samples would be theoretically allocated between REF 6.25 and REF 3.1

N.A. Not applicable

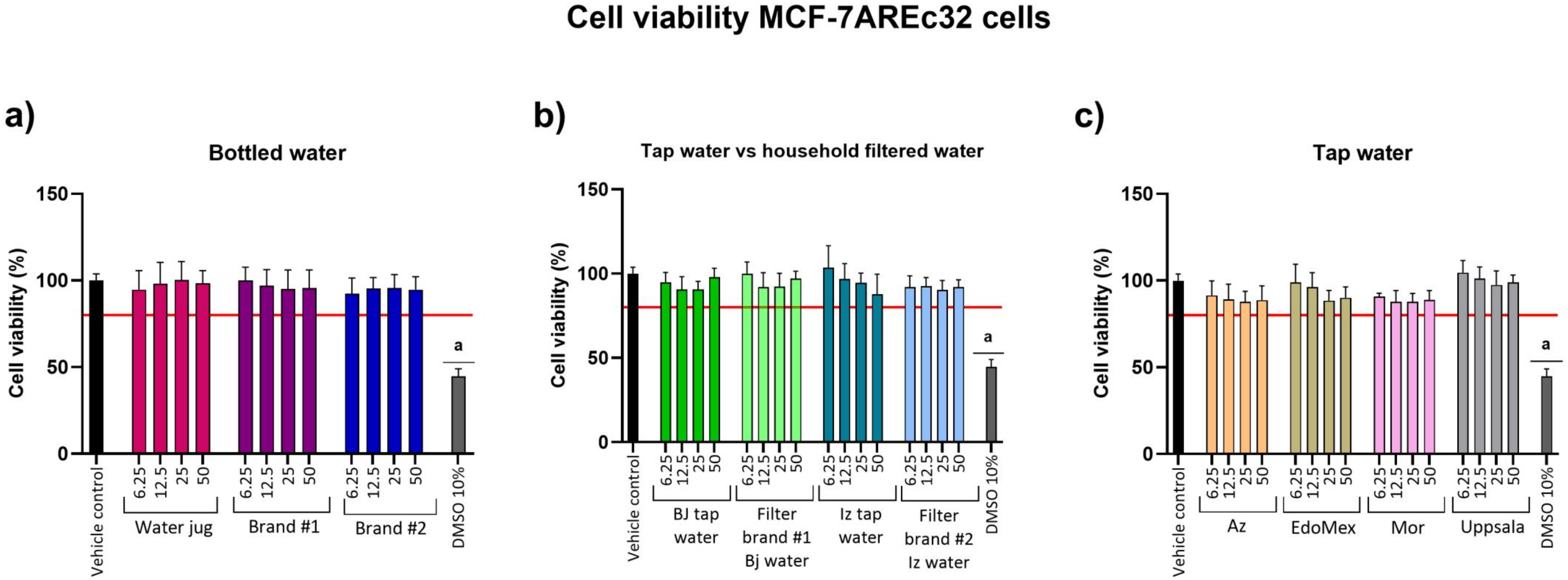

Figure S2. Cell viability of MCF-7 AREc32 cells exposed to water samples and DMSO 10% as positive control for 24 hrs. Samples were categorized as **a)** bottled water (water jug, brands #1 and #2), **b)** tap water and filtered tap water using two different household filter brands in two different demarcations in Mexico City (Benito Juárez and Iztapalapa) and **c)** tap water from different sources (Azcapotzalco demarcation in Mexico City, State of Mexico and Morelos states and reference sample from Uppsala, Sweden). Sample concentrations are expressed as REF (6.25-50). Data is presented as mean ± SD, n= 8 (two independent experiments with four technical replicates per concentration) and values lower than 80% viability are presented as **a**.

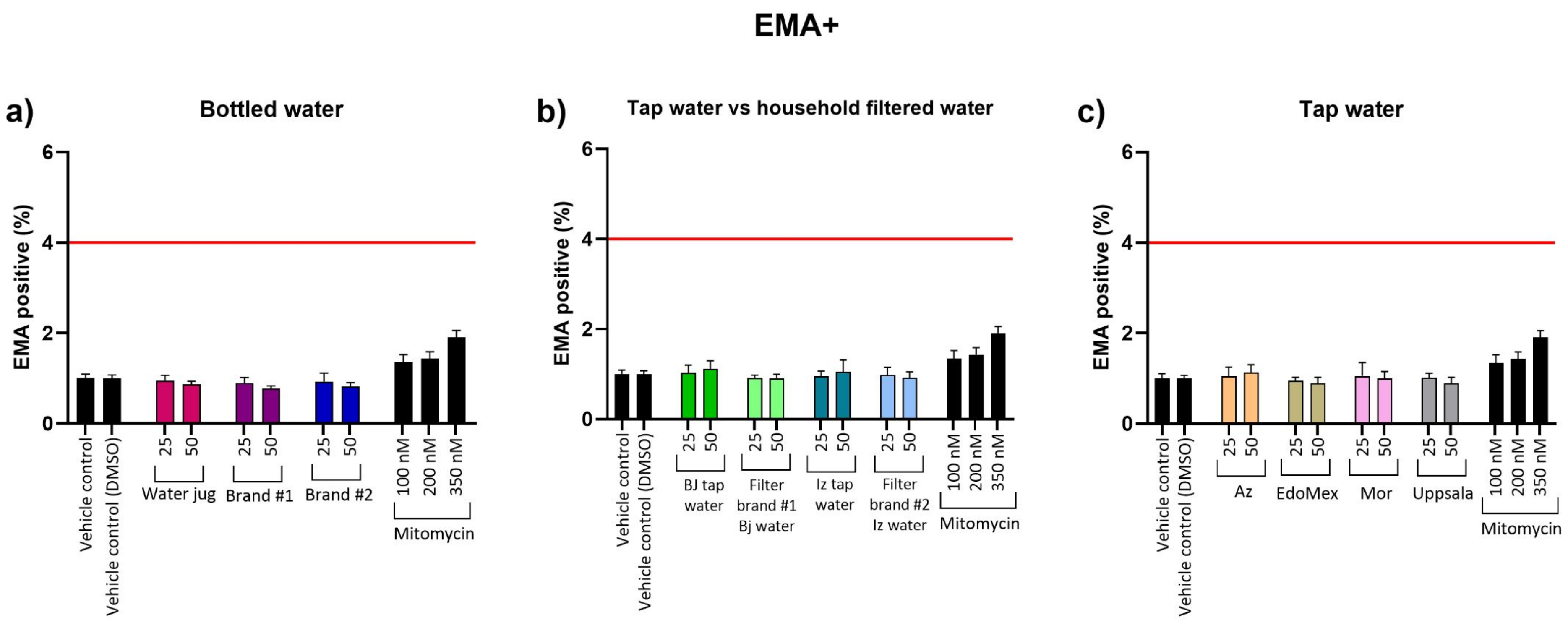

Figure S3. Cell viability (EMA+) of TK6 cells exposed to water samples and Mitomycin C (100, 200 and 350 nM) for 24 hrs. Samples were categorized as **a)** bottled water (water jug, brands #1 and #2), **b)** tap water and filtered tap water using two different household filter brands in two different demarcations in Mexico City (Benito Juárez and Iztapalapa) and **c)** tap water from different sources (Azcapotzalco demarcation in Mexico City, State of Mexico and Morelos states and reference sample from Uppsala, Sweden). Sample concentrations are expressed as REF (25 & 50). Data is presented as mean ± SD, n= 8 (two independent experiments with four technical replicates per concentration) and the line represents the cut-off limit in fold change (≥ 4) for defining cytotoxicity.

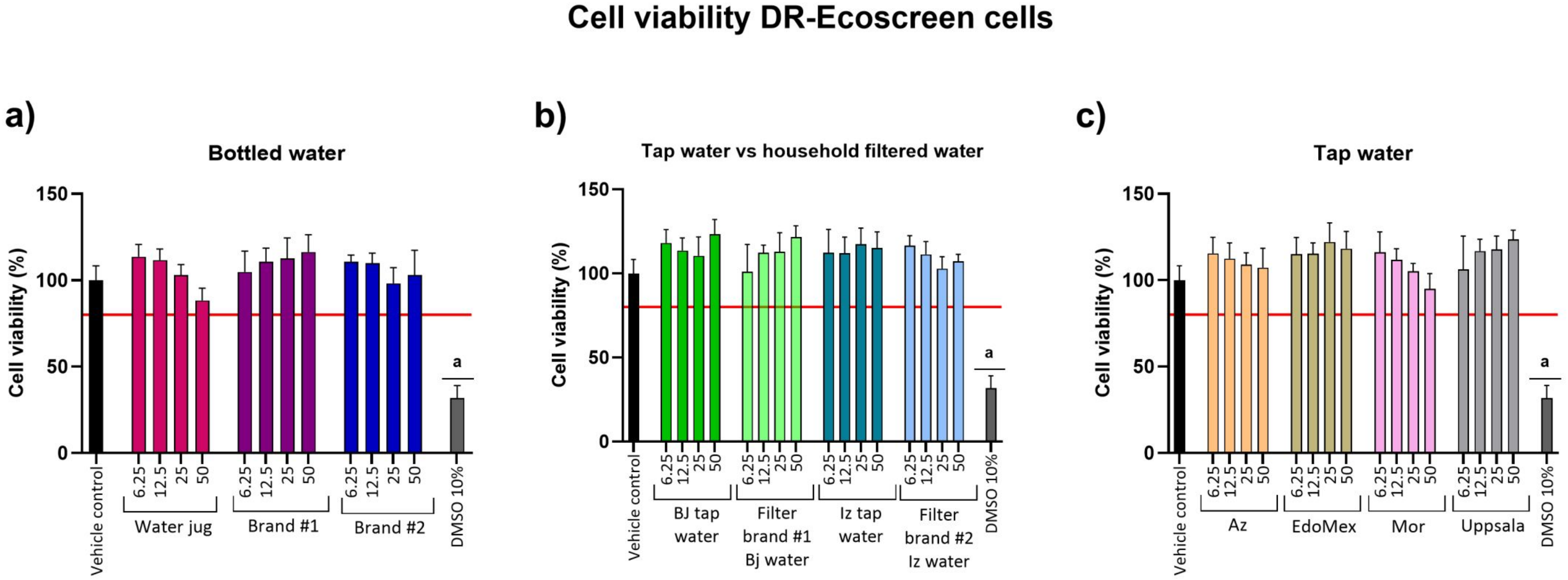

Figure S4. Cell viability of DR-EcoScreen cells exposed to water samples and DMSO 10% as positive control for 24 hrs. Samples were categorized as **a)** bottled water (water jug, brands #1 and #2), **b)** tap water and filtered tap water using two different household filter brands in two different demarcations in Mexico City (Benito Juárez and Iztapalapa) and **c)** tap water from different sources (Azcapotzalco demarcation in Mexico City, State of Mexico and Morelos states and reference sample from Uppsala, Sweden). Sample concentrations are expressed as REF (6.25-50). Data is presented as mean ± SD, n= 8 (two independent experiments with four technical replicates per concentration) and values lower than 80% viability are presented as **a**.

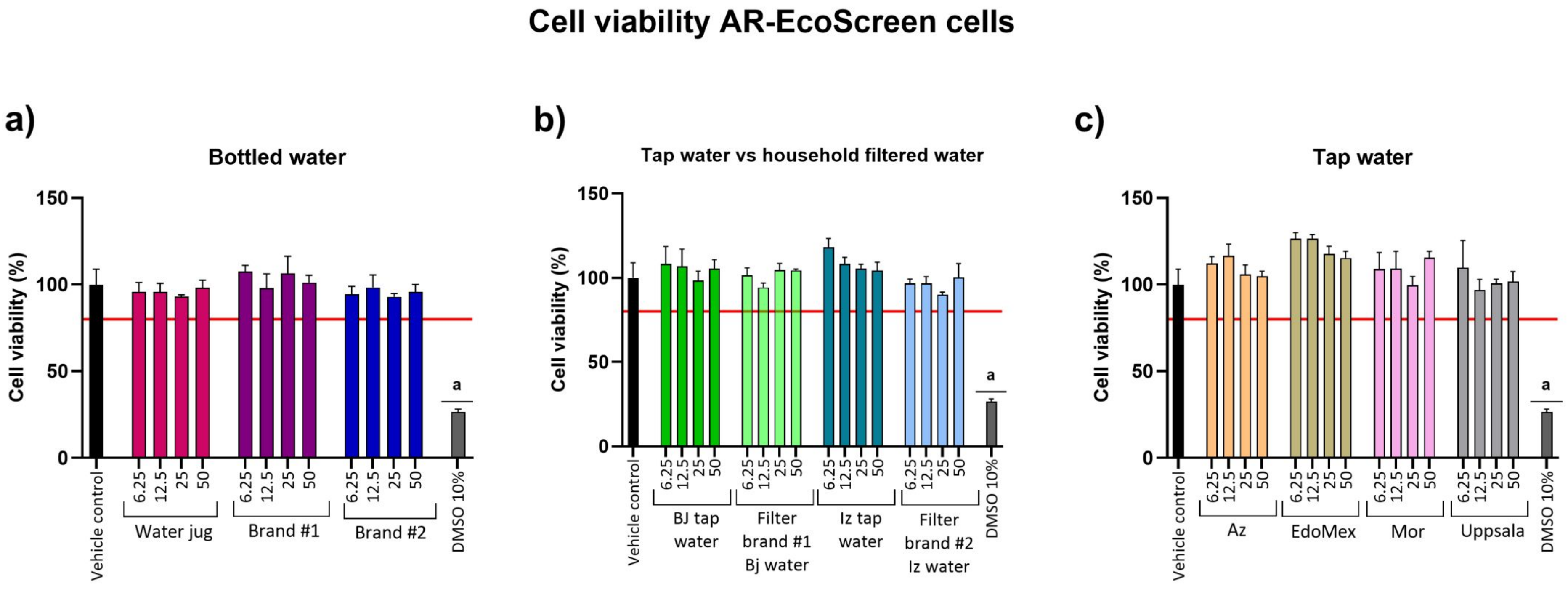

Figure S5. Cell viability of AR-EcoScreen cells exposed to water samples and DMSO 10% as positive control for 24 hrs. Samples were categorized as **a)** bottled water (water jug, brands #1 and #2), **b)** tap water and filtered tap water using two different household filter brands in two different demarcations in Mexico City (Benito Juárez and Iztapalapa) and **c)** tap water from different sources (Azcapotzalco demarcation in Mexico City, State of Mexico and Morelos states and reference sample from Uppsala, Sweden). Sample concentrations are expressed as REF (6.25-50). Data is presented as mean ± SD, n= 8 (two independent experiments with four technical replicates per concentration) and values lower than 80% viability are presented as **a**.

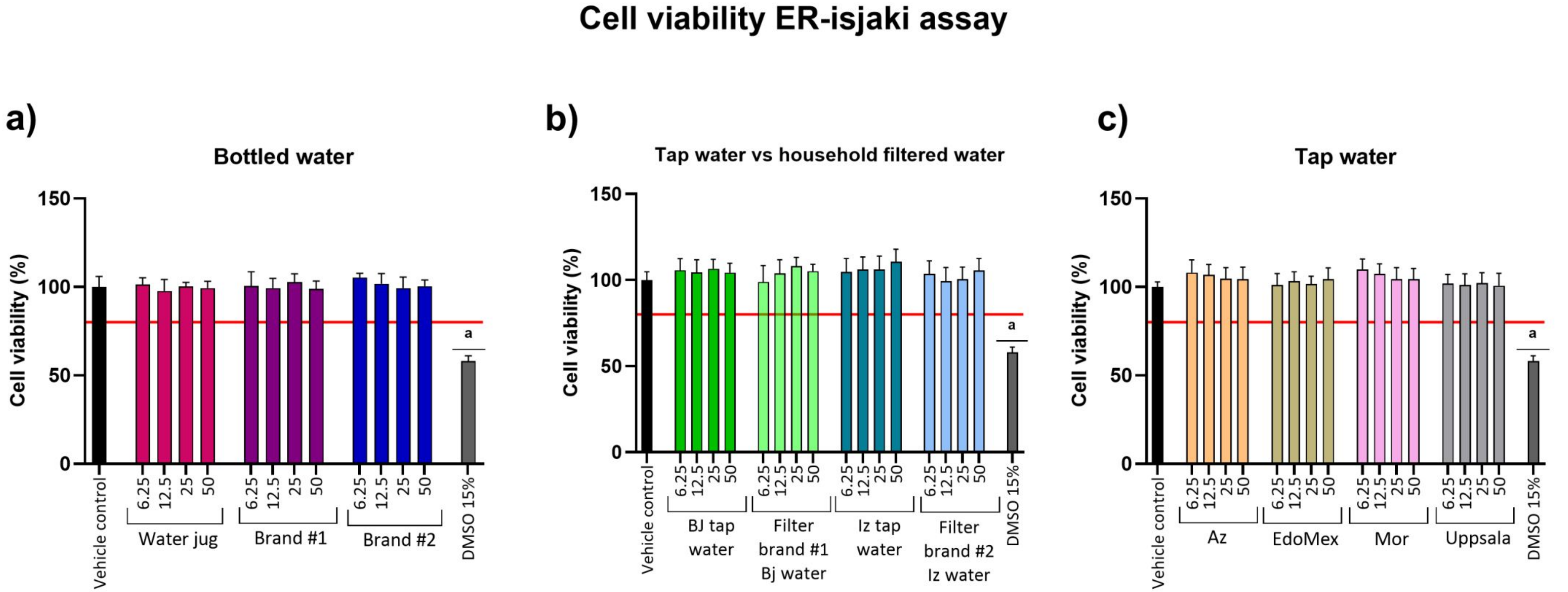

Figure S6. Cell viability of in MCF-7 cells transiently transfected with 5 ng per well of the *pNL2.3-ERE* plasmid (ER-isjaki assay) and exposed to water samples and DMSO 15% as positive control for 24 hrs. Samples were categorized as **a)** bottled water (water jug, brands #1 and #2), **b)** tap water and filtered tap water using two different household filter brands in two different demarcations in Mexico City (Benito Juárez and Iztapalapa) and **c)** tap water from different sources (Azcapotzalco demarcation in Mexico City, State of Mexico and Morelos states and reference sample from Uppsala, Sweden). Sample concentrations are expressed as REF (6.25-50). Data is presented as mean ± SD, n= 9 (two independent experiments with three or six technical replicates per concentration) and values lower than 80% viability are presented as **a**.

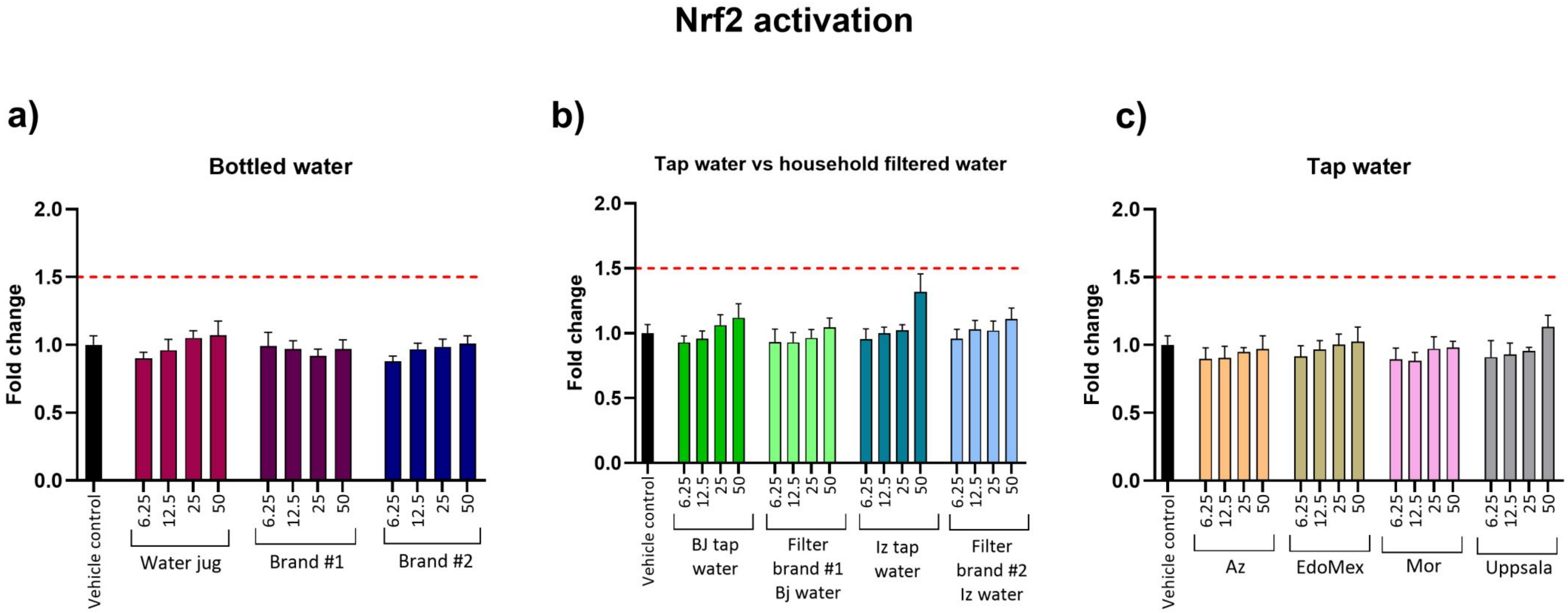

Figure S7. Activation of the Nrf2 transcription factor indicative of oxidative stress in MCF-7 AREc32 cells exposed to water samples for 24 hrs. Samples were categorized as **a)** bottled water (water jug, brands #1 and #2), **b)** tap water and filtered tap water using two different household filter brands in two different demarcations in Mexico City (Benito Juárez and Iztapalapa) and **c)** tap water from different sources (Azcapotzalco demarcation in Mexico City, State of Mexico and Morelos states and reference sample from Uppsala, Sweden). Sample concentrations are expressed as REF (6.25-50). Data is presented as mean ± SD, n= 8 (two independent experiments with four technical replicates per concentration) and the dotted line represents the bioactivity cut-off limit in fold change (1.5) for the assay.

Genotoxicity (MN+)

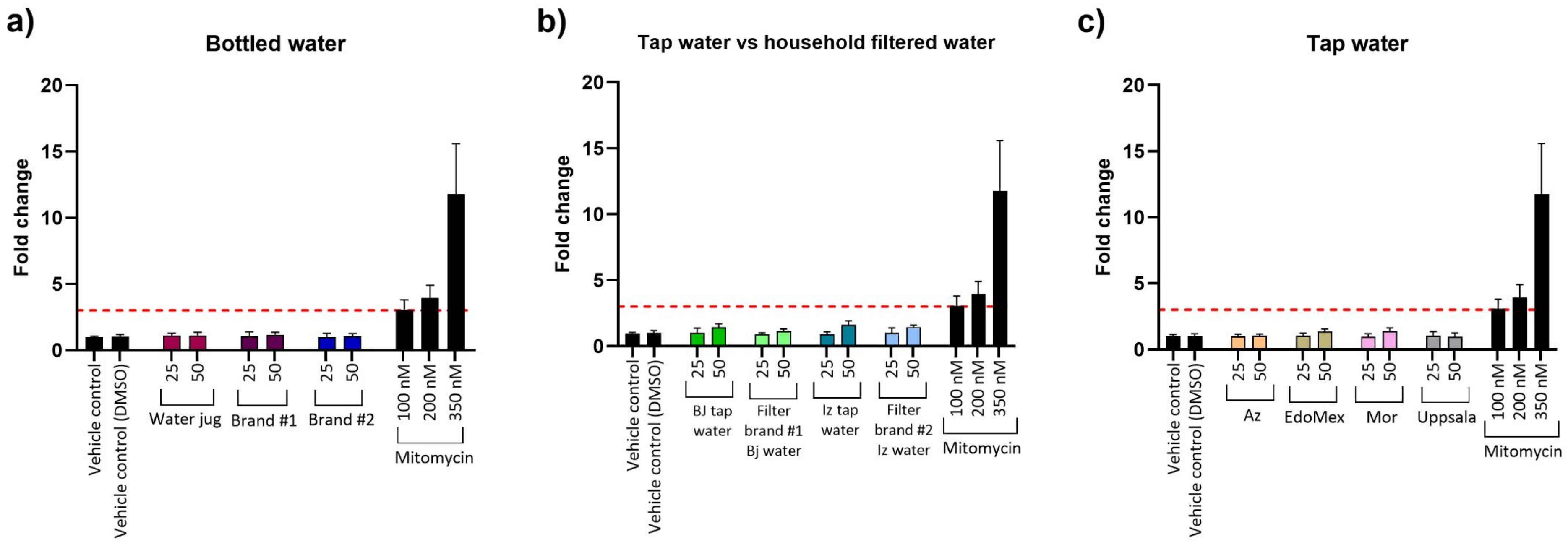

Figure S8. Genotoxicity assessment (micronuclei formation) in TK6 cells exposed to water samples and Mitomycin C (100, 200 and 350 nM) for 24 hrs. Samples were categorized as **a)** bottled water (water jug, brands #1 and #2), **b)** tap water and filtered tap water using two different household filter brands in two different demarcations in Mexico City (Benito Juárez and Iztapalapa) and **c)** tap water from different sources (Azcapotzalco demarcation in Mexico City, State of Mexico and Morelos states and reference sample from Uppsala, Sweden). Sample concentrations are expressed as REF (25 & 50). Data is presented as mean ± SD, n= 8 (two independent experiments with four technical replicates per concentration) and the dotted line represents the cut-off limit in fold change (3) for the assay.

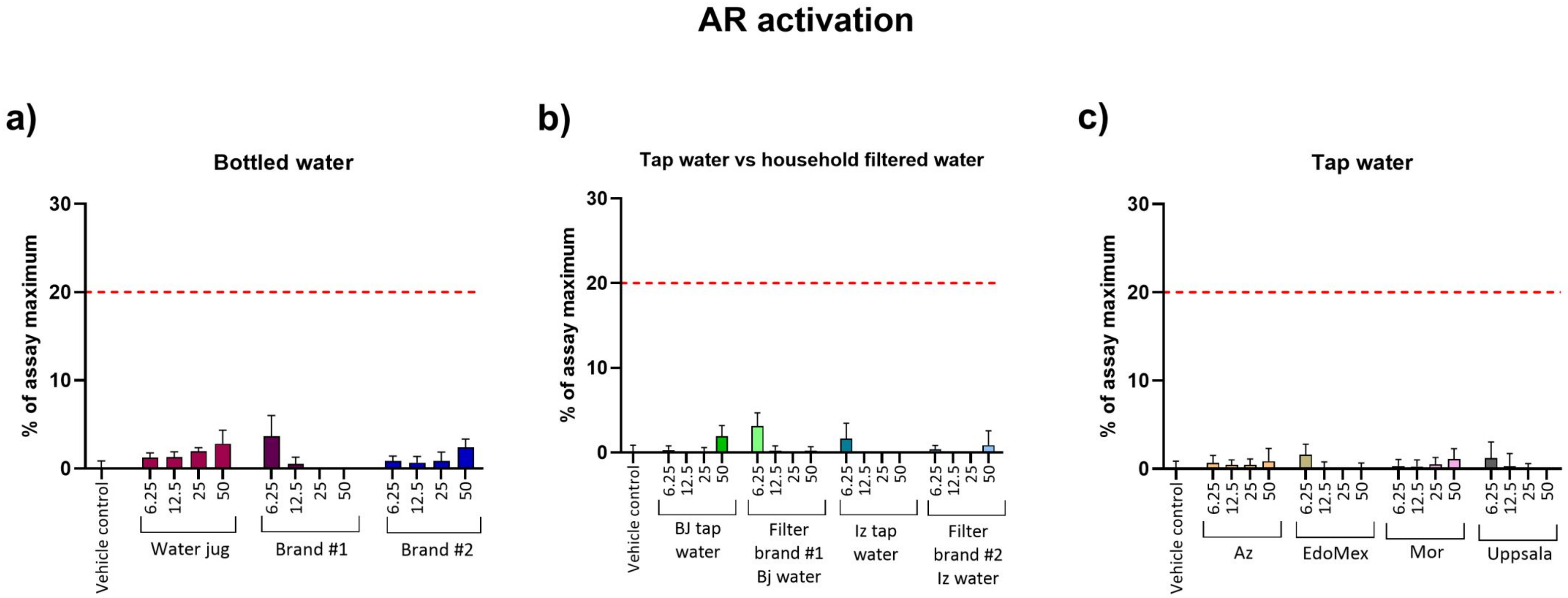

Figure S9. Activity of the AR in AR-EcoScreen cells exposed to water samples for 24 hrs. Samples were categorized as **a)** bottled water (water jug, brands #1 and #2), **b)** tap water and filtered tap water using two different household filter brands in two different demarcations in Mexico City (Benito Juárez and Iztapalapa) and **c)** tap water from different sources (Azcapotzalco demarcation in Mexico City, State of Mexico and Morelos states and reference sample from Uppsala, Sweden). Sample concentrations are expressed as REF (6.25-50). Data is presented as mean ± SD, n= 8 (two independent experiments with four technical replicates per concentration) and the dotted line represents the bioactivity cut-off limit in % of assay maximum (20% effect).
